# The Least Squares Method as a Tool for Assessment of the Stroke Parameters and Velocity in Monofin Swimming

**DOI:** 10.3390/mps8010019

**Published:** 2025-02-17

**Authors:** Marek Rejman, Paweł Szkudlarek

**Affiliations:** Department of Swimming, Wroclaw University of Sport and Health Sciences, 51-612 Wroclaw, Poland

**Keywords:** finswimming, kinematics, modeling

## Abstract

This study explores the application of the Least Squares Method to analyze and model the kinematic parameters in monofin swimming, focusing on stroke rate, stroke length, and the amplitudes of joint displacements at the hip, knee, and ankle. The primary aim is to evaluate whether this method provides an objective and diagnostic tool for assessing monofin swimming techniques. Three elite monofin swimmers were evaluated under a progressive fatigue test. Results indicated that the stroke rate increases velocity by 0.95, 0.23, and 0.96 units (for the estimated models respectively). Optimized stroke length (0.01–0.12 units) also significantly correlates with velocity improvements. Joint amplitude reductions, particularly at the hip and ankle, enhanced propulsion by minimizing drag. This study highlights the Least Squares Method as a diagnostic tool for optimizing swimming techniques, with potential applications in performance training.

## 1. Introduction

In sports based on cyclic and propulsive movements (including swimming), time and velocity are the factors determining performance. Simultaneously, they provide basic and objective criteria for its evaluation [1]. In swimming, due to water resistance, the result obtained in a race—much more than in other sports—depends on the level of the swimmer’s technique. Therefore, biomechanical methods of technique assessment have become an indispensable element in controlling the training of technical skills. The modeling of movement structures is one of these methods and plays an important role in the process of the objective evaluation of movement. This role arose from the fact that technical error is defined as the execution of a movement not in accordance with its pattern (model) [2]. From this perspective, biomechanical modeling giving information on the dynamic and kinematic structure of movement supports the process of developing the required technical skills [3].

Monofin swimming has gained further recognition as an independent competitive sport. Over the past decade, global participation in finswimming has increased by more than 30%, with a growing number of national federations adopting the discipline (Confédération Mondiale des Activités Subaquatiques, https://www.cmas.org/finswimming/how-to-regulations.html, accessed on 15 February 2025). While finswimming is not yet included in the Olympic Games, CMAS continues to advocate for its recognition at the highest level of competition. Beyond finswimming, monofin techniques are widely used in the technical training of competitive swimmers to enhance propulsion during underwater phases. Studies indicate [4] that refining underwater dolphin kicks with monofins can lead to a 5–10% reduction in race times for short-distance events, underscoring their significance in performance optimization.

Monofin swimming, characterized by its unique propulsion mechanics, requires precise technical execution for optimal performance. Unlike traditional swimming, the reliance on a single fin amplifies the impact of kinematic variables such as stroke rate, stroke length, and joint amplitudes.

In studies on the development and optimization of monofin swimming, various forms of biomechanical modeling have been presented. Among them are modeling based on the analysis of water flow over the surface of the monofin (Computational Fluid Dynamics (CFD)) [5,6], conceptual simulation models [7], and mathematical models [8,9]. Artificial Neural Networks (ANNs) have also been used as a modeling tool in the construction of the functional model of the monofin swimming technique [10,11]. However, quantitative tools that assess these parameters systematically remain scarce. However, a review of the available literature has identified only one study that successfully applied the Least Squares Method (LSM), for analyzing both aerobic and anaerobic parameter metrics in monofin swimming [12]. No studies were found here in the field of biomechanics. This study investigates LSM as a novel approach for evaluating monofin swimming techniques to provide objective insights for performance optimization.

LSM is based on Legendre’s postulate, which reads as follows: “The most likely value of a series of measurements taken under the same conditions is the value at which the calculated deviations from these results, after raising to the second power and summing, give the smallest possible figure” [13]. The approach with this method is, therefore, to estimate the structural parameters of the model in order to explain the behavior of one variable with the behavior of other variables, where the relation between the response variable and explanatory variables is linear. Using LSM in modeling derives from econometrics. The econometric model is a system of hypotheses, formulated in a mathematical way (either in the form of an equation or a system of equations), which show the basic relations existing between the actual phenomena under consideration [14]. As previously mentioned, numerous methods for evaluating and modeling the monofin swimming technique are employed in practice.

This study does not aim to evaluate LSM in comparison to other methods but rather to propose this quantitative tool as an alternative due to its practical applicability in technical training of monofin swimming. The LSM proves to be particularly effective in analyzing relationships between variables, which is essential in the context of tools used for optimizing processes [15], as demonstrated here by changes in the kinematic parameters of the monofin swimming technique. An additional advantage of LSM in practical applications lies in its solid mathematical foundation, which ensures the predictability and stability of results, particularly with small databases [15]. LSM is characterized by its simplicity and accessibility [16]. It allows for direct data acquisition and does not necessitate specialized staff for the development of analysis tools and or interpretation of the results. As such, the structure of LSM is more straightforward to understand, facilitating analysis and enabling rapid conclusions. Simple models, such as those based on LSM, are easier to implement and interpret in practical applications. Another advantage of LSM is its computational efficiency [16]; LSM does not require substantial computational resources, making it a more affordable and accessible option for smaller research institutions and coaching centers.

At the same time, the application of theoretical knowledge in practical training and the biomechanical interpretation of the technique model must be objective and fully understandable to coaches and swimmers. These demands are generally met through the accepted evaluation of the biomechanical parameters of the swimming stroke (stroke rate, stroke length, and the amplitude of propulsive movements). The parameters mentioned, as a measure of the quality of a swimming technique, present a broad biomechanical and physiological justification. This results from the fact that the characteristics of movement are directly linked to the level of fatigue increase during swimming. The evaluation of the monofin swimming technique through the analyses of stroke parameters has not been carried out as often as in traditional swimming. However, research based on the comparison of the efficiency of propulsion while swimming barefoot, and with different types of fins [17,18,19] allows the transfer of most of the depicted dependencies to monofin swimming.

A high level of technical ability will allow the swimmer to maintain the parameters of the stroke at a stable level over the entire distance covered [20], limit the energy cost [21], and generally reduce the impact of increasing fatigue, thus forming conditions for the attainment of maximum swimming speed [20,21,22,23,24,25,26,27] An evaluation of the quality of a technique clearly underscores the role of stable stroke rate and stable stroke length in relation to swimming speed [21,28,29,30,31]. The amplitude of movements can be treated as a parameter that characterizes performance in monofin swimming [16,19,32]. The postulate that the optimization of amplitude in order to gain maximal swimming speed has been formulated. In this context, the stroke parameters analyzed emerged as the measure of the key criterion of performance [33,34] and the efficiency of swimming [35], i.e., striving to increase the swimming speed while minimizing fluctuation of the intracycle velocity. The usefulness of the parameters of the swimming stroke (stroke rate, stroke length, and the amplitude of propulsive movements) in the assessment of the quality of the monofin swimming technique seems to be proven. Therefore, these parameters can be treated also as tools for verifying the diagnostic value of the model identified.

This study is embedded in issues related to the modification of the motor behavior of a swimmer equipped with a monofin, towards the effective and efficient employment of it as a source of propulsion. Its purpose is to analyze the kinematic parameters of the monofin movements (stroke rate, stroke length, and amplitude of displacement of the hip, knee, and ankle joints), in the context of determining, through modeling with LSM, its impact on increasing swimming velocity. The aim was pursued by verifying the hypothesis that modeling with LSM can be an objective tool for assessing the monofin swimming technique. The hypothesis was verified in two stages: (1) through the description and interpretation of the relationship between the stroke parameters under consideration and the swimming velocity with the monofin, and (2) by resolving whether the interpretation of the relationships identified on the basis of modeling the monofin swimming technique with LSM has an objective and diagnostic dimension in a statistical and empirical sense.

## 2. Materials and Methods

Three, highly skilled male swimmers, belonging to the elite of monofin swimming, voluntarily took part in the research. The characteristics of the participants are given in Table 1.

The swimmers performed a test, swimming at progressively faster speeds. The trial distances were divided into 3 × 300 m sections. The swimmers rested for 60 s after each section of the trial distance. The test previously used in monofin swimming analysis [11] was adapted from training in traditional swimming, where it is conventionally used [1,36]. The test was chosen with the intention of simulating stress conditions and to analyze the biomechanical parameters of swimming technique during increasing fatigue. Swimmers swam individually on the water’s surface, in a short course pool. They swam with their own monofins, which met their individual preferences and were the fins they usually used for competitions. In this way, recording was accomplished eliminating the factors that disturb swimmers’ swimming technique, which arise from the use of unknown propulsive equipment.

**Figure 1:**
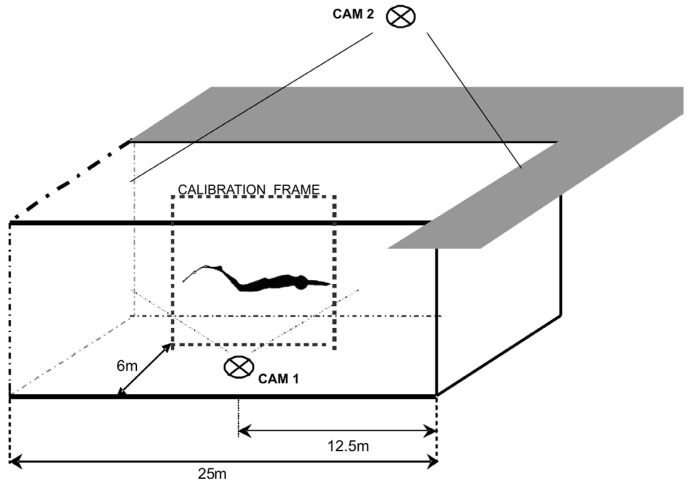
General description of experimental setup.

**Figure 2:**
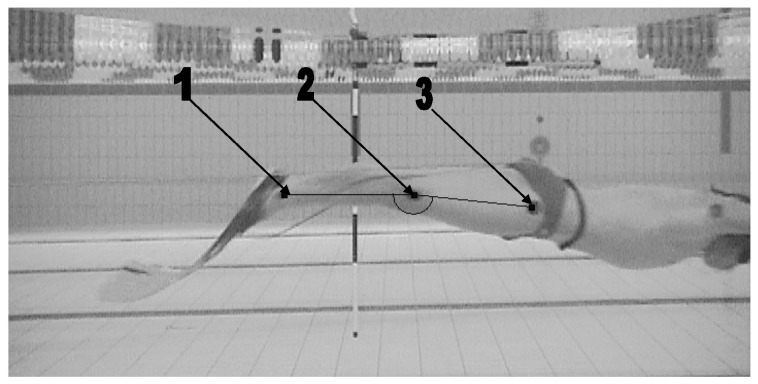
Illustration of the placement of the markers on the swimmers’ leg—on the axis of ankle joint (1), knee joint (2), and hip joint (3).

In order to collect parameters describing the monofin swimming technique, all swimmers were recorded with an underwater camera. A digital camera (GoProHero 4, GoPro, United States) was located in a stable position in the middle of the pool. Based on the assumption that the movement of the swimmer and monofin act in a sagittal plane [37] the location of the camera was chosen to hold the largest possible image of the swimmers in frame, over more than the entire stroke (6 m distance from the calibration frame (2 m × 2 m)) [10] (Figure 1). The axis of the lens was perpendicular to the objects recorded. The recording frequency was 50 Hz. Markers that allowed the tracking of displacement of the transverse axes of the ankle, knee, and hip joints were located on both legs [38] (Figure 2).

**Figure 3:**
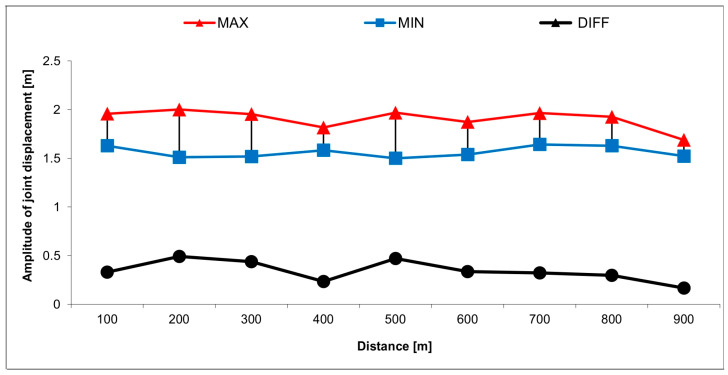
The explanation of estimation of the amplitude of displacement of the leg joints (hip, knee, and ankle) as the distance (DIFF) between deepest (MIN) and highest (MIN) position of the ankle in the stroke.

Kinematic analyses of the leg segment displacements were conducted using the SIMI Motion Analysis System v.10.2 (SIMI Reality Motion Systems GmbH, Germany). Random strokes (recorded over the last 25 m from all nine of the 100 m intervals of the 3 × 300 m test distance), were chosen for analysis. The results were obtained in the form of temporal signals for the amplitude of displacement of the joints mentioned above (AMP_ankle_, AMP_knee_, and AMP_hip_, defined as a distance between the deepest and highest position of the ankle in the stroke) (Figure 3). The average horizontal velocity was also estimated. The center of the swimmer’s body mass was computed using SIMI software v.10.2, in which the marker-based method was adapted [39] by assuming the displacement of the hip joint marker placed on the swimmer’s body [40] is tracked.

The strokes performed by each swimmer were recorded from above the water (Figure 1) at the same measures of distance (the last 25 m of all nine 100 m intervals). For the estimation of mean stroke length (SL—distance swum in one stroke) the strokes performed over a 15 m distance, well visible to the camera’s lens (5 m from each wall of the pool) were recorded. The number of strokes were read, directly from the digital image, by two independent viewers. For estimation of the mean stroke rate (SR—stroke number per minute), SIMI software was also employed. The data regarding the stroke parameters was collected in conjunction with underwater recording of the kinematic data.

The model, developed on the basis of LSM, reflected a linear relationship between the stroke rate, stroke length, amplitude, and swimming velocity. The average horizontal swimming velocity was taken as the response variable, whose variability is explained by the model. The explanatory variables used to explain this variation were the above-mentioned parameters characterizing the kinematics of the swimming stroke. There were 30 models created, 10 for each subject. All the models for a single swimmer described—in various configurations—the relationship between the parameter and the velocity, starting with the relationship of all the parameters to the velocity, and ending with the relationship of a random single parameter to the velocity. Probing the results of each tested swimmer, the three selected were those who obtained the highest speed and who in the finest manner realized the assumptions of the progressive swimming test. Among the models constructed for each competitor, there was one selected which in the best way showed the linear character of the tested relations, that is the model describing the relationship of all the parameters to the velocity:

***y_i_* = *β*_0_ + *β*_1_*x*_1*i*_ + *β*_2_*x*_2*i*_ + … + *β_k_x_ki_* + *ε***,  *i =* 1, 2, …, *n*

where:

*y*—response variable;

*x*—explanatory variables;

*β*—estimators of the structural parameters searched in the model;

*ε*—random error;

*k*—the number of estimated parameters;

*n*—size of sample;

*i*—number of the observation.

In order for the estimators of the structural parameters of the model introduced above to show the statistical characteristics, the following conditions should be fulfilled:

1. The explanatory variables are non-random and their values are treated as constants in the repeated attempts. (This assumption is derived from the application of LSM in the experimental research). The amplitude of the axis displacement of the examined leg joints, the stroke rate, and the stroke length do not meet this assumption in the swimming conditions. However, the repealing of this assumption does not result in significant loss of the estimators’ properties, provided that the explanatory variables and random components are independent, and this fact can be accepted.
2. Expected values of the random components are zero, i.e., ***E*(*ε_i_*) = 0** for *i* = 1, 2, …, *n*. (which means that interferences represented by the random components have a tendency toward mutual reduction, i.e., random components can be either positive or negative, so the expected value of random components ***E*(*ε_i_*)** equals zero).
3. The variances of the random components ***ε_i_*** are fixed, i.e., ***D*^2^(*ε_i_*) *= σ*^2^** for *i* = 1, 2, …, *n*. (assumption refers to the fact that the noise variance does not depend on the number of observations, i.e., the value ***D*^2^(*ε_i_*)** is always constant, therefore, it is accepted to denote it with ***σ*^2^** symbol for all tests).
4. Random components ***ε_i_*** and ***ε_j_*** are independent for ***i*** **≠ *j* = 1, 2, …, *n*** (This assumption says that dins of the model are not correlated between different observations, i.e., that there is no direct link between the errors in the given samples).
5. Each of the random components ***ε_i_*** has a normal distribution (This assumption stems from the fact that in practice the errors cumulating in the random component have very often an approximately normal distribution. This assumption is not necessary while employing LSM, but with its fulfillment, the statistical inference is much more easily conducted).
6. The sample size is greater than the number of estimated parameters, i.e., ***n* > *k* + 1** (where *n* = 9 and *k* = 5), and between the observation vectors of explanatory variables, there is no linear relation. (These assumptions refer to the numerical problems of determining the estimators. If these assumptions are met, the least squares estimators can be unambiguously determined. This means that the parameters ***β_i_*** obtained by this method are the only possible parameters.)

Data analyzed in this study had an approximately normal distribution and met the above assumptions (Table 2).

The estimators ***β*** were calculated using the GRETL program (Gnu Regression, Econometrics and Time-Series Library http://gretl.sourceforge.net/, accessed on 15 February 2025). Econometric coefficients were used to make inferences, and interpreted with respect to empirical research. The following interpretation was adopted: the evaluation of the impact of the explanatory variables on the response variable is as follows: in the model ***y_i_* = *β*_0_ + *β*_1_*x*_1*i*_ + *β*_2_*x*_2*i*_ + … + *β_k_x_ki_*** the evaluation ***b_j_*** of the structural parameter of ***b_j_***, ***j* = 1, 2, …, *k***, defines how the average level of the response variable ***y*** is increased (when ***b_j_*** < 0) or decreased (when ***b_j_*** > 0) when the explanatory variable ***x_j_***, is increased by one unit, assuming that values of other explanatory variables haven’t changed.

## 3. Results

The analysis of the coefficient values (Table 3) indicates that the constructed models exhibit linear relationships between the explanatory variables and the mean horizontal swimming velocity (response variable). The estimated effects of the explanatory variables on velocity are as follows:

A one-unit increase in stroke rate increases velocity by the following:
  - 0.95 units (Model B), 0.23 units (Model M), and 0.96 units (Model A).
A one-unit increase in stroke length increases velocity by the following:
  - 0.01 units (Model B), 0.02 units (Model M), and 0.12 units (Model A).
A one-unit reduction in hip displacement amplitude (hip joint axis) increases velocity by the following:
  - 0.67 units (Model B), 0.60 units (Model M), and 0.76 units (Model A).
A one-unit reduction in knee displacement amplitude (knee joint axis) increases velocity by the following:
  - 0.51 units (Model B), 0.50 units (Model M), and 0.43 units (Model A).
A one-unit reduction in foot displacement amplitude (ankle joint axis) increases velocity by the following:
  - 0.75 units (Model B), 1.43 units (Model M), and 0.53 units (Model A).

To assess the explanatory power of these models, the eta-squared coefficient (η^2^) was calculated. The results indicate the following:

- For Model B, η^2^ ≈ 0.988, meaning that the model accounts for 98.8% of the variance in swimming velocity. This suggests a very good fit, with the model explaining nearly all observed variability in the data.
- For Models M and A, η^2^ = 1, indicating that these models explain 100% of the variance in the dependent variable, suggesting a perfect fit.

These results highlight the strong predictive power of the models, with stroke rate and joint displacement amplitudes having the most substantial effects on swimming velocity. The near-perfect or perfect η^2^ values suggest that the selected explanatory variables significantly contribute to explaining the variance in the estimated models.

The results obtained on the basis of econometric coefficients analysis (Table 3) indicate a proportional model relationship between the stroke rate and the average swimming velocity. This relationship is very clear for two of the constructed models. The proportional relationships between the extension of the stroke length and swimming velocity increment have a similar value for all the models but are much lower compared to those of the stroke rate. It was also shown that the limited amplitude of hip and foot displacement clearly favors an increase in the average swimming velocity; whereas the effect of limiting the amplitude of the knee displacement on the swimming velocity is significantly lower.

**Figure 4:**
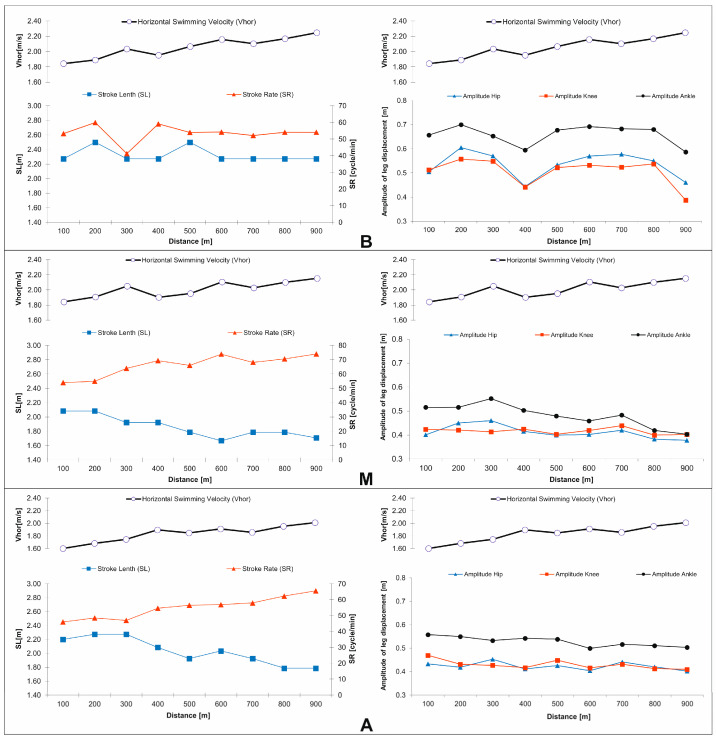
Graphs illustrating the division of the stroke parameters (stroke rate (SR) and stroke length (SL)) and the amplitudes of displacement of hip, knee, and ankle, compared with average horizontal swimming velocity (V_HOR_) registered on all nine of the 100 m intervals of the test distance performed by particular swimmers researched. (B, M, A)—the initials of the swimmers tested.

The results based on the real data recorded indicated that in the group of swimmers studied, swimming velocity increased when the stroke rate increased and the stroke length became shorter (Table 3). The dependencies depicted by the model showed that both of these stroke parameters increased proportionally to the increase in velocity. Therefore, an analysis of the trials performed by particular swimmers (Figure 4) should be carried out. When attention is paid to the last 300 m section, where all the swimmers swam fastest, stabilization of the stroke parameters (particularly so in the case of stroke length, while less so in regards to stroke rate) is visible.

The results describing the amplitude of the leg segment displacement examined both in the model (Table 3) as well as in the empirical (Figure 4) aspect appear to be similar. The increase in swimming velocity takes place as a consequence of the limiting of hip and knee displacement (limitation in the range of thigh movements) and knee and foot displacement (limitation in the range of calf movement). The displacement in the hip and knee are almost identical (substantial limitations of thigh movements) and significantly smaller in comparison with the amplitude of foot displacement. The limiting of knee and foot (shin) displacement is located in the intermediate range of values in comparison to the displacement of other joints (segments) and implies a limited up-and-down knee displacement.

The interpretation of the statistical analysis presented in Table 3 draws attention to the fact that in each of the constructed models, *p*-values for the explanatory variables (stroke rate, stroke length, and the amplitude of hips, knees, and feet displacements) exceed the required significance level (0.05), and, therefore, the calculated coefficients are statistically significant. The results of Student’s *t*-test allow the inference that the models reflect a statistically significant relationship between the average horizontal velocity of monofin swimming, stroke rate, stroke length, and amplitude of the hips, knees, and feet displacement. The values of the coefficients of determination R-squared and adjusted coefficients of determination R-squared (considered high if above 0.3, and indicates the suitability of each of the models to the reported data). Small values of the sum of squared residuals and the values of standard errors of residuals indicate that the average deviation of the theoretical (model) values from the empirical (real) values is very small. Finally, as mentioned above, the very high η^2^ values suggest that the selected explanatory variables are highly relevant (however, in practical applications it is crucial to consider the potential risk of overfitting, especially in the case of small datasets). In light of this, one can assume that each of the models is well suited to the conditions of the test performed. Diagnostic values of the models constructed have been confirmed statistically. Thus, the basic conditions for applying LSM as the objective assessment tool of the monofin swimming technique seem to be fulfilled.

## 4. Discussion

The verification of the hypothesis that the modeling of LSM enables one to objectively assess the monofin swimming technique was performed on the basis of evidence resulting from empirical studies that confirm the statistical interpretation of the results and determine a diagnostic dimension of the constructed models. The analysis of the constructed models indicates the existence of a relationship between swimming speed and stroke rate, stroke length, and amplitude of the hip, knee, and foot movement, assuming that the change of each tested parameter of the stroke has an impact on the speed of swimming.

The ability to swim the maximal distance within one stroke (stroke length) has an enormous effect on the achievement of maximal swimming speed, together with the simultaneous ability to repeat these strokes using the maximal frequency (stroke rate). This crucial criterion of the efficiency of swimming propulsion [1,36] was indicated by the models estimated. Nonetheless, with respect to the results based on direct recording, the mentioned interaction in relation to the swimming speed has been not confirmed. This fact can be understood as undermining the diagnostic value of the models constructed in the sense of the assumptions stated in this study. However, employing the knowledge based on the practical experience of depreciation of the models estimated seems to be unreasonable.

Nomura and Shimoyama [26] determined that insufficient technical skill, or a change in swimming technique resulting from fatigue, are often causes of increased stroke rate and shortened stroke length. Other research has established that depending on the race distance, at a given velocity, stroke rate should be reduced and stroke length lengthened, in order to limit the workload and reduce energy requirements [17,18]. This tendency is visible in the results describing the last (fastest) 300 m part of the test distance (Figure 3). A trend toward stabilizing the stoke parameters is also shown. The phenomenon of the stabilization of stroke length occurred more visibly than the stabilization of stroke rate (which had a tendency to increase). The suggestion that an increase in swimming velocity may be achieved by increasing stroke rate, maintaining a stable stroke length [28,41], or by both of these strategies [29] was formulated in other studies. The above tendencies also correspond to the results of research by Nomura and Shimoyama [26] as well as Toussaint et al. [42], who observed that as the result of increasing fatigue over the final part of the test distance (the last (fastest) 300 m), swimming efficiency dropped. The ability to maintain a stable stroke length, regardless of the increasing effects of fatigue, is a measure of the individual level of the technical skill of the swimmer [21,24,25]. In the context mentioned, the result of this study seems to give rise to the need to investigate criteria for maximization of monofin swimming speed in the optimization of the stroke parameters (stroke rate, stroke length, and amplitude). The direction of this optimization might be designated from the patterns of fish locomotion [43], which hint that the best conditions for maintaining maximal swimming speed are created by maintaining a propulsive movement amplitude, at a more or less constant level, while simultaneously increasing stroke rate [19,41].

The juxtaposition of the results related to the registered amplitude of displacement of the joint hip, knee, and ankle joint axis (Figure 3) with the results generated using LSMs (Table 3) validates in the empirical dimension the relationship resulting from the models. The results suggest that reducing the amplitude of displacement in the considered joints enhances the increase in swimming velocity, which is confirmed by the postulate of the reduction in the degree of freedom of the biomechanical chain of the leg segments [44].

The dominant role of foot displacement in generating propulsion identified in this study was revealed in the results based on the construction of a neural network [11], as well as research on the factors favoring the maintenance of high intracycle velocity in monofin swimming [44]. Another study [45] suggests that controlling the foot movement is the most difficult part of the movement structure of monofin swimming, in comparison to the other segments of the leg. Therefore, the proper displacement (amplitude) of the feet plays, from an educational point of view, the most significant role in the process of generating propulsion. The feet are perceived as the last, active (fully controlled by the swimmer) element in the biomechanical chain of the swimmer’s body and are directly linked to the transfer of torque to the surface of the monofin [19,20,44,45]. Among others, the limiting of plantar flexion during downbeat, allows one to avoid the significant overload of the muscle–ligament apparatus at the ankle joint, which causes pathology [46].

The limiting of the hip amplitude with the aim of increasing monofin swimming velocity, which is suggested by the results obtained, leads to the stiffening of the torso and consequently to a reduction in the drag resulting from too large an undulating movement [47]. Limiting the degree of freedom in the hip joint also restricts the downward displacement of the knee acting as a documented factor of minimization of the drag (generated on the front surface of the bending thigh) [47].

The impact of reducing the amplitude of the knee displacement on the increase in swimming velocity estimated by the models is smaller than the amplitude of hip displacement. At the same time, it has been proven that limiting thigh movement (hip-knee) and calf movement (knee-foot) implies a reduction in up-and-down knee displacement. The lower leg flexion (upward movement) is a key part of the stroke that on one hand, allows the use of the monofin as a source of propulsion, and on the other hand, impacts the minimization of the drag that is a result of the lower leg movements according to the direction of swimming [47].

The analysis of the amplitude in the segments of the leg displacement during monofin swimming seems to create an analogy with the mechanism illustrating the most effective and most efficient form of movement in water being tuna-like [46]. When referring to the propulsion movements performed by a human equipped with a monofin to the propulsive movements of a tuna, the efficient and effective propulsion will be conditioned by the undulating movements of legs, so that the extent of each segment movement increases in the optimum range from the hips towards the feet (with immobilization of the head and shoulder girdle, as well as the torso and hips). Several studies [18,40] have demonstrated that greater amplitude leads to a larger effective cross-sectional area, and possibly induces more drag. Therefore, an adjustment in kick frequency at the optimal level of constant amplitude usually leads to achieving the highest monofin swimming velocity [18,19].

In comparison to more complex methods such as ANN and CFD, LSM does not necessitate the use of complicated programming or training processing [16]. This characteristic renders the results obtained with this method, such as regression functions, straightforward to comprehend, implement, and interpret. This simplicity is particularly relevant for diagnostic purposes in sports, where rapid analysis, immediate feedback for athletes and coaches, and application of findings into technical training—such as monofin swimming—are priorities. The simplicity of LSM is also evident in its clearly defined mathematical foundations (principles of error minimization), which reduce the risk of subjective errors stemming from imprecise model assumptions. The resulting predictability and stability of the results are crucial for reliable analysis of technical parameters (including monofin swimming), where precision has a direct impact on performance [15]. In this context, it can be supposed that LSM is particularly suited for research aimed at optimizing movement techniques, as it proves in analyzing relationships between variables. Furthermore, its diagnostic validity in small datasets [15] makes it preferable over other methods for modeling based on the inherently limited groups of elite athletes. The practical value of LSM in the analysis of sports technique also stems from its accessibility under conditions of limited computing power [16] This is particularly advantageous for small research institutions and coaching centers, as it eliminates the need for advanced and costly equipment. As mentioned above, LSM has been used for modeling the relationships between aerobic parameters (e.g., VO₂max and ventilatory threshold), anaerobic parameters (e.g., power derived from countermovement jumps and force-velocity tests), and swimming performance. The results of these analyses have shown strong correlations, including several anthropometric characteristics [12]. This example, in the context of the above arguments, suggests that LSM could function as a valuable tool for biomechanical analysis in the monofin swimming technique and evaluation of its impact on performance.

The description of the results obtained and the interpretation of the relationship between stroke rate, stroke length, and average velocity in monofin swimming have extensive factual justification. However, the objective diagnostic dimension of the modeling of the distribution of stroke rate and stroke length to achieve maximum swimming velocity with a monofin, based on LSM, seems to be limited. First, coefficients of the variation calculated for the stroke rate values are on the border for fulfilling the assumptions of this method. Secondly, there is a lack of foundation necessary to resolve how the statistical dimension of the constructed models takes into account the “empirical” conditions that affect the results achieved by the swimmers, e.g., the impact of fatigue on the modification of the distribution strategy of stroke rate and stroke length. The interpretation of parameters describing the amplitude of leg segment movements appears to meet the criteria for reliability and objectivity. However, according to some authors [18], the diagnostic value of amplitude as a factor in determining an increase in monofin swimming velocity is significantly lower compared to stroke rate or stroke length. The reliability of the results obtained and the postulate of their practical application are not diminished by the small sample size. This is because the athletes studied represent an elite group of swimmers, i.e., a highly specific population that actually performs exemplary technical models. Their exclusivity limits the pool of participants for research [48], while rigorous training schedules further restrict their availability [49]. In this context, it is worth reiterating the adequacy of using LSM as a modeling method, as it provides stable results in the case of small datasets [15]. The high specificity of the results for the research context means that findings from small samples are often more representative of elite populations than those obtained from larger, heterogeneous groups [50]. In order to resolve the above limitations, it would be advisable to extend the experiment by including the collection and analysis of objective markers of energy expenditure and validate their applicability with larger groups of monofin swimmers across skill levels.

Limiting the analysis to the displacement of leg segments in the hip, knee, and ankle joints may also raise doubts. As previously mentioned, reducing the displacement in the considered joints enhances the increase in swimming velocity [47]. In the same study, this was confirmed by the postulate of reduction in the degrees of freedom of the biomechanical chain of the leg segments (including feet). Furthermore, propulsion movements performed by a human equipped with a monofin can be compared to the propulsive movements of a tuna fish [47], where efficient propulsion is conditioned by the undulating movements of legs such that the extent of each segment movement increases in the optimum range from the hips. These arguments should sufficiently explain the limiting of the analysis of body segment displacement in this study to thighs and shanks. The movement of these body segments strongly determines the amplitude of the leg/fins—a key variable in defining the objective of the study.

## 5. Conclusions

The analysis of stroke rate, stroke length, and amplitude of displacement of the leg segments while monofin swimming (from the point of view of indicating the model dependencies that condition maximum swimming speed) proved that the average swimming velocity increases in proportion to increases in the stroke rate and stroke length. The increase in swimming velocity occurs also as a consequence of a reduction in hip and knee displacement (limiting the range of thigh movement) as well as knee and foot movement (limiting the range of calf movement). These dependencies confirmed the usefulness of the Least Squares Method for modeling the monofin swimming technique. This method provides a reliable framework for assessing the monofin swimming technique, offering valuable insights into stroke rate, stroke length, and joint displacement amplitudes. By quantifying these parameters, the method supports performance optimization and technical refinement. Future studies should validate these findings with larger samples and investigate their integration into athlete training regimens.

## Figures and Tables

**Table 1:** Characteristics of monofin swimmers participating in the study.

Participant	Age [years]	Body High [m]	Body Mass [kg]
I	15	1.75	70
II	16	1.71	64
III	15	1.63	64
VC	0.04	0.03	0.05

Note: VC—coefficient of variation.

**Table 2:** Basic statistics illustrating the absolute differences within the structural parameters of the constructed models.

Subject		Amplitude Hip	Amplitude Knee	Amplitude Ankle	Stroke Length	Stroke Rate	Horizontal Swimming Velocity
	Mean	0.53	0.51	0.66	2.32	53.40	2.05
B	SD	0.05	0.06	0.04	0.10	5.29	0.13
	VC	0.10	0.11	0.06	0.04	0.10	0.07
	Mean	0.41	0.42	0.48	1.86	66.08	2.00
M	SD	0.03	0.01	0.05	0.15	7.35	0.11
	VC	0.07	0.03	0.10	0.08	0.11	0.05
	Mean	0.42	0.43	0.53	2.03	54.99	1.83
A	SD	0.02	0.02	0.02	0.19	6.78	0.13
	VC	0.04	0.05	0.04	0.09	0.11	0.07

Note: SD—standard deviation; VC—coefficient of variation.

**Table 3:** This statistical report contains coefficients of models designated under The Least Squares Method, which reflect a linear relation between average swimming velocity and stroke rate (SR) stroke length (SL), and the amplitude of displacement of the leg segments in the hip joint (AMPhip), the knee joint (AMPknee), and the ankle joint (AMPankle).

	Paremeter	Model B	Model M	Model A
Coefficient	Const	1.47	0.54	2.67
	Stroke Length (SL)	0.01	0.02	0.12
	Stroke Rate (SR)	0.95	0.23	0.96
	Amplitude Hip (AMP_hip_)	−0.67	−1.6	−0.76
	Amplitude Knee (AMP_knee_)	−0.51	−0.50	−0.43
	Amplitude Ankle (AMP_ankle_)	−0.75	−1.43	−0.53
*p*-value (<0.05)	Const	0.32	0.64	0.52
	Stroke_Length (SL)	0.97	0.93	0.76
	Stroke Rate (SR)	0.13	0.57	0.39
	Amplitude Hip (AMP_hip_)	0.29	0.10	0.59
	Amplitude Knee (AMP_knee_)	0.57	0.48	0.63
	Amplitude Ankle (AMP_ankle_)	0.47	0.17	0.97
t-ratio	Const	1.18	0.62	0.93
	Stroke_Length (SL)	0.03	0.10	0.38
	Stroke Rate (SR)	2.04	0.79	1.40
	Amplitude Hip (AMP_hip_)	−1.27	−6.23	−0.75
	Amplitude Knee (AMP_knee_)	−0.62	1.04	−0.64
	Amplitude Ankle (AMP_ankle_)	0.82	3.45	−0.04
Mean Dependent Variable		1.65	1.57	1.41
Standard Deviation Dependent Variable		0.1	0.05	0.13
Sum Squared Residual		0.01	0	0
Residual Standard Error		0.06	0.01	0.04
R-squared		0.84	0.99	0.98
Adjusted R-squared		0.59	0.95	0.88
Eta-squared (η^2^)		0.988	1	1

Note: Const—a model constant; (B, M, A)—the tested monofin swimmers.

## Data Availability

Data are available upon reasonable request from the corresponding author.
